# CATP-6, a *C. elegans* Ortholog of ATP13A2 PARK9, Positively Regulates GEM-1, an SLC16A Transporter

**DOI:** 10.1371/journal.pone.0077202

**Published:** 2013-10-09

**Authors:** Eric J. Lambie, Pamela J. Tieu, Nadja Lebedeva, Diane L. Church, Barbara Conradt

**Affiliations:** 1 Department of Cell and Developmental Biology, Ludwig-Maximillians-University, Munich, Planegg-Martinsried, Germany; 2 Department of Biological Sciences, Dartmouth College, Hanover, New Hampshire, United States of America; 3 Parkinson's Center, Dartmouth Hitchcock Medical Center, Lebanon, New Hampshire, United States of America; 4 CIPS^M^ Center for Integrated Protein Science, Ludwig-Maximilians-University, Munich, Germany; University of North Carolina, United States of America

## Abstract

In previous work, we found that gain-of-function mutations that hyperactivate GEM-1 (an SLC16A transporter protein) can bypass the requirement for GON-2 (a TRPM channel protein) during the initiation of gonadogenesis in *C. elegans*. Consequently, we proposed that GEM-1 might function as part of a Mg^2+^ uptake pathway that functions in parallel to GON-2. In this study, we report that CATP-6, a *C. elegans* ortholog of the P5B ATPase, ATP13A2 (PARK9), is necessary for *gem-1* gain-of-function mutations to suppress the effects of *gon-2* inactivation. One possible explanation for this observation is that GEM-1 serves to activate CATP-6, which then functions as a Mg^2+^ transporter. However, we found that overexpression of GEM-1 can alleviate the requirement for CATP-6 activity, suggesting that CATP-6 probably acts as a non-essential upstream positive regulator of GEM-1. Our results are consistent with the notion that P5B ATPases govern intracellular levels of Mg^2+^ and/or Mn^2+^ by regulating the trafficking of transporters and other proteins associated with the plasma membrane.

## Introduction

P-type ATPase transporters are widespread within the genomes of both prokaryotes and eukaryotes, and include such well-studied proteins as the Na^+^, K^+^-ATPase, and the sarcoplasmic/endoplasmic reticulum Ca^2+^ pump (SERCA) [1], [2]. Eukaryotic P-type ATPases have been classified into five groups, P1-P5 [3], with the P5 subfamily the least well understood. The P5 ATPases are represented in plants, animals and fungi [4] and they have been divided into two subcategories, P5A and P5B based on amino acid similarity and structural organization. Furthermore, differences between the signature PPxxP motif within M4 suggest that P5A and P5B ATPases are likely to recognize different transport substrates [5]. Despite their deep evolutionary conservation, the biological functions and transport substrates of the P5 ATPases remain uncertain. In this paper, we will focus on the P5B ATPases.

The *S. cerevisiae* genome encodes a single P5B ATPase, Ypk9, which is localized to the vacuole membrane and has been suggested to transport Mn^2+^ into the vacuole [6], although no direct demonstration of this activity has been reported. In *Dicytostelium discoideum* the sole P5B ATPase, Kil2, is expressed on the phagosomal membrane and is required for Mg^2+^-dependent killing of ingested *Klebsiella*; this has prompted the suggestion that Kil2 pumps Mg^2+^ into the phagosome [7].

The *C. elegans* genome contains three P5B ATPase genes: *catp-5*, *catp-6* and *catp-7*, which are likely derived from a single ancestral gene that was present at the time of origin of the nematode phylum (the genomes of many other nematodes, e.g., Ascaris and Brugia, contain only a single P5B ATPase gene; WormBase WS238). The Caldwell group identified CATP-6 in a high-throughput screen for factors that prevent aggregation of human alpha-synuclein heterologously expressed in *C. elegans* body muscles [8]. Subsequently, CATP-6 was shown to be able to partially substitute for Ypk9p in *S. cerevisiae*, implying that it might be able to act as a Mn^2+^ transporter [6]. CATP-5 localizes to the apical brush border of *C. elegans* intestinal epithelial cells [9]. Inactivation of *catp-5* confers resistance to the toxic polyamine analog, norspermidine, and also impairs uptake of polyamines by the intestinal cells; therefore, Heinick et al. (2010) have suggested that CATP-5 might be a polyamine transporter. No characterization of CATP-7 has been reported.

The mouse and human genomes encode four P5B ATPases, ATP13A2-ATP13A5 [10]. Potential biological functions have been suggested for each of these except ATP13A5. ATP13A3/AFURS1 was found to be upregulated in senescent cultured human parenchymal kidney cells, suggesting an association with cellular aging [11]. A chromosomal rearrangement that disrupts ATP13A4 was found to be associated with autism, suggesting a potential role in neural development [12]. Vallipuram et al. (2010) subsequently reported that ATP13A4 localized to the endoplasmic reticulum (ER) when expressed in COS-7 cells, and that overexpression of ATP13A4 resulted in increased cytosolic levels of free Ca^2+^ [13].

ATP13A2 (also known as PARK9) has been the most intensively studied human P5B ATPase, since mutations in this gene lead to juvenile onset Parkinson disease [14], [15]. ATP13A2 localizes to lysosomal membranes when expressed in human tissue culture cells [15], suggesting that it might perform a function comparable to that of Ypk9p and CATP-6, e.g., sequestering Mn^2+^ in a vesicular compartment, and thus protecting dopaminergic neurons from alpha- synuclein aggregation and cellular dysfunction [6], [8]. A series of recent reports have suggested that ATP13A2 function is important for general aspects of lysosome function [16] and neuronal physiology [17]. Based on high-throughput interactome studies, Usenovic et al. suggested that ATP13A2 directly interacts with components of the vesicular trafficking machinery [18], and Covy et al. [19] have proposed that ATP13A2 is required for lysosomal import of an unspecified physiological cofactor.

In this paper, we describe our identification of *catp-6* as a locus that genetically interacts with the *gon-2* and *gem-1* loci of *C. elegans*. *gon-2* encodes a TRPM cation channel protein that is required for Mg^2+^ uptake by the intestinal epithelial cells, and likely also by the somatic gonadal precursor cells, Z1 and Z4 [20], [21]. Inactivation of *gon-2* leads to a severe gonadogenesis defect, and in some animals Z1 and Z4 fail to undergo any divisions [22]. *gem-1* encodes an SLC16A major facilitator transporter protein [23]. Although the founding members of the SLC16A family are well-characterized as monocarboxylate transporters, the identities of the transport substrates for most proteins in this family are not known [24]. In previous work, we found that hypermorphic gain-of-function alleles of *gem-1* are able to bypass the requirement for *gon-2* in gonadogenesis [23]. We also showed that expression of *gem-*1 within Z1 and Z4 is sufficient to rescue the gonadogenesis defect of *gon-2* mutants. Therefore, we proposed that hyperactivation of GEM-1 leads to increased activity of a parallel Mg^2+^ uptake system within the gonadal precursors. In this paper, we demonstrate that CATP-6 activity within Z1 and Z4 is necessary and sufficient in order for *gem-1(gf)* to suppress *gon-2(lf)*. Furthermore, we find that overexpression of GEM-1 is able to bypass the requirement for CATP-6 during gonadogenesis. Therefore, we propose that CATP-6 normally promotes the activity of GEM-1, which then acts to increase the level of free Mg^2+^ within the gonadal precursors.

## Methods

### Nematode culture and genetic manipulation

Nematodes were maintained on NGM plates with the *E. coli* strain AMA1004 [25] as food source. With the exception of SNP mapping experiments, all strains used were in an N2 Bristol background. The wild strain CB4856 was used for SNP mapping experiments, essentially as described by Jakubowski et al. [26]. Standard methods were used for strain constructions [27]. PCR, sometimes in conjunction with DNA sequencing, was done to validate genotypes as necessary.

### Molecular biology

Standard methods were used for DNA analysis and manipulation. Oligonucleotides were obtained from Eurofins. For large PCR products we used either Phusion or LongAmp DNA polymerase (New England Biolabs). We obtained a *catp-6::gfp* fosmid clone derivative of WRM067B_F08 from the *C. elegans* TransgeneOme project [28] and used this for transformation rescue and expression analyses. We obtained very similar results when we used *in vivo* recombination between fosmid WRM067B_F08 and a PCR fragment to generate a C-terminally tagged version of *catp-6* that lacked the *catp-6* 3′UTR. However, since expression from the TransgeneOme clone was typically brighter, we used this construct for all of the analyses detailed below.

### Isolation of *catp-6(lf)* mutations

Hermaphrodites of genotype *gon-2(q388); gem-1(gf)* were mutagenized with 50 mM EMS for 4 hrs [27]. F1 progeny were plated in groups of three in 12-well plates containing NGM solid medium seeded with *E. coli* and allowed to produce F2 progeny at 23.5°. Plates were then screened for wells that contained multiple gonadless individuals. In total, we screened approximately 3,000 mutagenized genomes. Fertile siblings were then cloned at 15°, and F3 progeny were subcloned and analyzed to identify derivatives that were homozygous for *catp-6(lf)*.

### Transgenic strains

Standard microinjection methods were used to generate and maintain transgenic animals. Plasmid pRF4 [29], which contains the dominant Roller marker, *rol-6(su1006)*, was used in all injection mixes at a concentration of approximately 150 μg/ml. Expression/rescue constructs were injected at a concentration of approximately 50 μg/ml.

The oligonucleotide primer pairs and templates used to generate DNA segments used for injection mixes were as follows:

o1594 (GGCCCCAAATAATGATTTTATTTTGCGGGTGGCGCACGACGC ) plus o1843 (aggtcgtcccgaatgttctg) were used to amplify the entire *catp-6* transcription unit, plus sequences flanking the 3′ UTR from the recombineerome fosmid. The underlined section of o1594 corresponds to the sequence from −23 to −7, relative to the initiation codon for *catp-6*. The first 25 nucleotides of o1594 provide homology for in vivo recombination with PCR-amplified promoter segments (see below). o1843 is identical to nucleotides +406 to +387 relative to the stop codon for *catp-6*.

o1587 (TCGCGTTAACGCTAGCATGGATCTCGAAGCTTGGGCTGCAGGTCGG ) plus o1588 (CAAAATAAAATCATTATTTGGGGCC TTGGGTCCTTTGGCCAATCC ) were used to amplify the *myo-3* promoter from pPD96.52 (Fire Lab 1995 Vector Kit). The underlined section of o1587 is a forward primer at the 5′ end of the promoter segment. The underlined section of o1588 is a reverse primer at the 3′ end of the promoter segment. The first 25 nucleotides of o1588 provide homology for recombination with the *catp-6::gfp* PCR product.

o1725 (AAGAGGTCCCGCTCCAACAAC) plus o1600 (CAAAATAAAATCATTATTTGGGGCC TTTGTAATTTGGAAGCTGGGAGGAATA ) were used to amplify the *ehn-3* promoter from wild type genomic DNA. o1725 is a forward primer at the 5′ end of the promoter. The underlined section of o1600 is a reverse primer at 3′ end of promoter. The first 25 nucleotides of o1600 provide homology for recombination with *catp-6::gfp*.

o1880 (ttgagccaatttatccaagtcc) and o1881 (CAAAATAAAATCATTATTTGGGGCC atcggtttggttggaagcgg) were used to amplify the *unc-119* promoter from pCFJ150 (Addgene plasmid 19329) [30]. o1880 is a forward primer at the 5′ end of the promoter, whereas o1881 is reverse primer that includes homology for recombination with *catp-6::gfp* as described above.

### Microscopy and imaging

Worms were mounted on 4% agarose pads for DIC and epifluorescence microscopy. In most cases, 10 mM levamisole was used to immobilize animals. Images were acquired using either a ) Zeiss Imager.M2 equipped with appropriate optics and a CCD camera connected to a PC running Time to Live (Caenotec) software, or b) a Zeiss Axioskop 2, equipped with appropriate optics and a PC running MetaMorph (Molecular Devices) software. Brightness and contrast of images was adjusted in Image J and/or PowerPoint.

### Assessment of gonad development in different genetic backgrounds

The presence of a vulva was used to determine whether animals had completed the initial set of gonadal cell divisions required to generate an anchor cell [31]. In most cases, vulva development was well-correlated with the ability of an animal to produce fertilized eggs (fertility); however, animals scored as fertile did not necessarily produce a normal number of viable progeny.

For Tables 1 and 2, L4 stage hermaphrodites were incubated overnight at the indicated temperature, then cut open in M9 buffer the next day in order to release eggs from the uterus. Eggs were then transferred by micropipette to seeded plates and raised at the indicated temperature until adult, at which point gonadogenesis and fertility were assessed.

**Table 1:** Effect of genotype on gonadogenesis at 20 degrees.

	Genotype	%Vulvaless	n
1	Wild type	0	>1000
2	*gem-1(0)*	0	>1000
3	*catp-6(0)*	0	>1000
4	*catp-6(0); gem-1(0)*	0	>1000
5	*gon-2(ts)*	0.5	1920
6	*gon-2(ts); catp-6(0)*	14.7	1475
7	*gon-2(ts); gem-1(0)*	64.0	753
8	*gon-2(ts); catp-6(0); gem-1(0)*	65.9	334
9	*gon-2(ts); catp-6(0); gem-1(dx66gf)*	21.2	391
10	*gon-2(ts); gem-1(dx66gf)*	0	>1000
11	*gon-2(ts); catp-6(0); gem-1(dx75gf)*	5.8	360
12	*gon-2(ts); catp-6(dx114); gem-1(dx69gf)*	7.6	303

Animals were raised and scored as described in Methods. *gem-1(0)* is *gem-1(bc364), catp-6(0)* is *catp-6(ok3473)*, *gon-2(ts)* is *gon-2(q388)*. Z test for two population proportions was used to assess signifcance (p<0.05) of differences between different values. Lines 1,2,3,4 and 10 are not significantly different from each other, but are significantly different from all other lines. Lines 5, 6, and 9 are significantly different from all other lines. Lines 7 and 8 are not signifcantly different from each other, but are significantly different from all other lines. Lines 11 and 12 are not significantly different from each other, but are different from all other lines.

**Table 2:** Effect of genotype on gonadogenesis at 23.5 degrees.

	Genotype	%Vulvaless	n
1	Wild type	0	>1000
2	*gem-1(0)*	0	>1000
3	*catp-6(0)*	0	>1000
4	*catp-6(0); gem-1(0)*	0	>1000
5	*gon-2(ts)*	98.6	1928
6	*gon-2(ts); catp-6(0)*	93.1	736
7	*gon-2(ts); gem-1(0)*	100	1453
8	*gon-2(ts); catp-6(0); gem-1(0)*	98.2	332
9	*gon-2(ts); catp-6(0); gem-1(dx66gf)*	88.6	134
10	*gon-2(ts); gem-1(dx66gf)*	0	>1000
11	*gon-2(ts); catp-6(0); gem-1(dx75gf)*	80	445
12	*gon-2(ts); gem-1(dx75gf)*	1.9^1^	325
13	*gon-2(ts); catp-6(dx114); gem-1(dx69gf)*	89.5	152
14	*gon-2(ts); gem-1(dx69gf)*	1.4^1^	367

Animals were raised and scored as described in Methods. Genotypes are as in Table 1. Z test for two population proportions was used to assess signifcance (p<0.05) of differences between different values. Lines 1,2,3,4 and 10 are not significantly different from each other, but are significantly different from all other lines. Lines 5 and 8 are not signifcantly different from each other but are significantly different from all other lines. Line 6 is not significantly different from line 13, but is significantly different from all other lines (but p = 0.046 for line 9). Line 7 is signficantly different from all other lines. Line 9 is significantly different from all but line 13. Line 11 is signifcantly different from all other lines. Lines 12 and 14 are not signifcantly different from each other, but are significantly different from all other lines.

1 Values from Kemp et al., 2009 [23].

For transgenic experiments, L4 or young-adult stage Rol animals were transferred to the restrictive temperature for *gon-2(q388)* (23.5°), and Rol progeny were then scored 3–5 days later, as they reached adult stage. Animals were scored as fertile if they had multiple fertilized eggs in the uterus. This represents a more stringent measure of successful gonad development than the presence/absence of a vulva. The vast majority of such animals had normal vulva development.

## Results

### Identification of *catp-6* mutations as suppressors of *gem-1(gf)*

**Figure 1:**
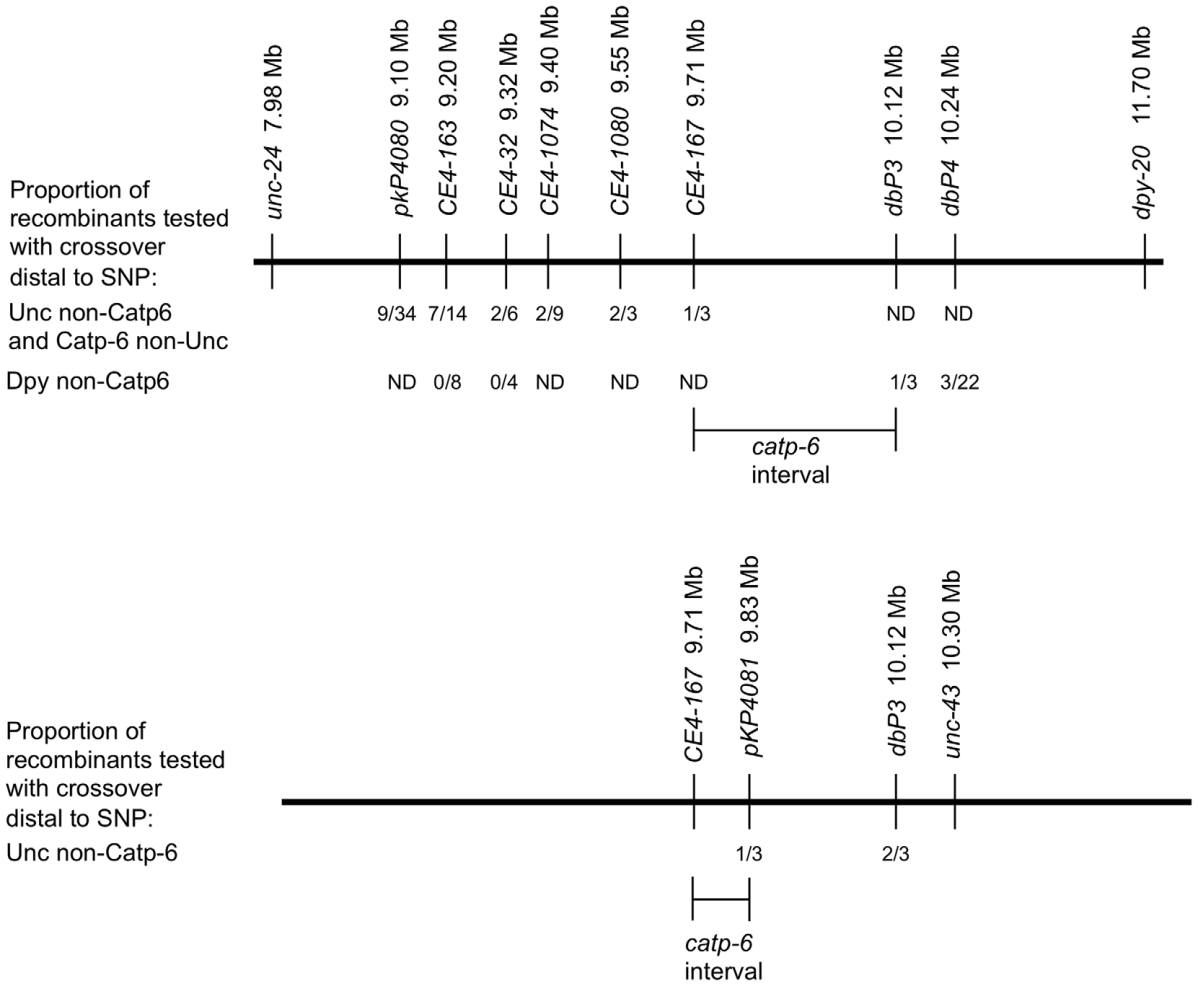
SNP mapping of *catp-6*. Recombinant progeny were analyzed from animals of three different genotypes: a) *gon-2(q388); unc-24(e138) catp-6(dx114)/CB4856; gem-1(dx66gf)*, b) *gon-2(q388); catp-6(dx114) dpy-20(e1282)/CB4856; gem-1(dx66gf)* (top panels), and c) *gon-2(q388); catp-6(dx114) unc-43(e408)/CB4856*; *gem-1(dx66gf)* (bottom panel). Not all SNPs were analyzed for each recombinant. Distances between SNPs, but not flanking markers, are to scale.

In an effort to identify null alleles of *gem-1*, we screened for EMS-induced mutations that reverted the *gem-1(gf)* phenotype, i.e., restored the Gon phenotype of *gon-2(q388); gem-1(dx66)* animals (see methods). Unexpectedly, four of the five mutations that we recovered did not map to the *gem-1* locus, but instead were located on chromosome IV. These four mutations (*dx110, dx112, dx113* and *dx114*), are all recessive and fail to complement each other. Since *dx114* appeared to be the most penetrant, we chose this allele for further characterization. First, through standard two and three-factor mapping, we determined that *dx114* is located between *unc-24* and *dpy-20* on chromosome IV. Next, through a series of SNP mapping experiments [26], we narrowed the location of *dx114* to a 120 kb interval (Figure 1). Since we obtained mutations in the *dx114* complementation group at a relatively high frequency, we sequenced the coding sequences of the largest predicted gene in this region, W08D2.5 ( =  *catp-6*). Consequently, we identified a single G -> A missense mutation associated with each of our mutant alleles.

**Figure 2:**
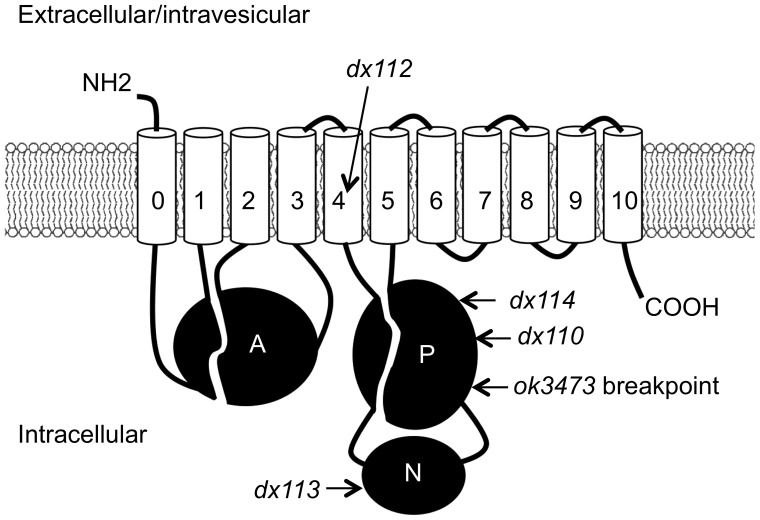
Locations of amino acids affected by mutant alleles of *catp-6* relative to conserved domains. The P (phosphorylation) domain, N (nucleotide binding) domain and A (actuator) domains are indicated. The transmembrane domains (M0-M10) are numbered 0-10. Relative locations of mutant alleles are indicated, including left breakpoint of *ok3473* deletion allele. Sizes of different domains are not strictly to scale.

According to WormBase (WS238), three different isoforms of CATP-6 are expected to be derived from the *catp-6* locus, each from a distinct mRNA. CATP-6a is 1256 aa in length, and has the typical structure of a P5B ATPase: eleven transmembrane segments (M0, plus M1-M10), with a relatively large cytoplasmic loop between M4 and M5 (Figure 2). The transcript for CATP-6c begins slightly 3′ relative to CATP-6a, resulting in a protein of 1207 aa that has the same overall structure. CATP-6b is significantly shorter, with a predicted length of 893 aa beginning just before M3. We have not attempted to determine whether each of these different isoforms is functional.

**Figure 3:**
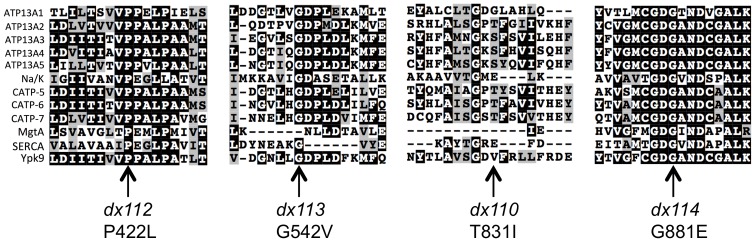
Locations of *catp-6* mutant alleles in comparison to other P-type ATPases. Alignments were done in TCoffee [35], followed by formatting in BoxShade. Accession numbers for protein sequences used in alignments are as follows: ATP13A1 NP_065143, ATP13A2 NP_001135445, ATP13A3 NP_078800, ATP13A4 NP_115655, ATP13A5 NP_940907, Na/K ATPase NP_001172014, CATP-5 NP_001024768, CATP-6 NP_001024768, CATP-7 NP_001023542, MgtA WP_020898295, SERCA NP_777613, Ypk9 NP_014934.

Three of the four mutant alleles that we identified affect residues situated within the large cytoplasmic domain between M4 and M5 (Figures 2 and 3). *dx113* converts a highly conserved glycine in the sequence LHGDP to a valine; this glycine is predicted to be situated between the first two helices of the N domain, and is immediately adjacent to residues that interact with Mg^2+^/ATP [32]. Therefore, *dx113* is likely to interfere with nucleotide binding. *dx114*, converts an invariant glycine in the middle of the sequence GDGAN to a glutamate. This glycine is within one of the essential Mg^2+^/ATP binding sites, so this mutation is also expected to disrupt nucleotide binding [32]. *dx110,* the third cytoplasmic mutation converts a threonine in the sequence GPTFA to an isoleucine; this residue is neither highly conserved nor expected to be in close proximity to the nucleotide binding site, but its alteration might disrupt the structure/function of the P domain. sThe sole mutation that affects one of the transmembrane domains, *dx112*, converts the highly conserved VPPALP sequence within M5 to VLPALP. Since this sequence is predicted to create the substrate binding pocket [33], *dx112* is expected to alter or severely impair transport activity.

As further verification of gene identity, we obtained the *C. elegans* Knockout Consortium allele, *catp-6(ok3473)*, and determined that it defines a 934 bp deletion within *catp-6*. Both of the endpoints of *ok3473* are located within exons, but since the junction results in a reading frame shift the last correctly coded amino acid is Ser765 (CATP-6a numbering); a stop codon occurs after 60 incorrectly coded amino acids (Figure 2). *ok3473* is thus expected to be a null allele; not only would the mRNA encode a protein lacking more than half of the transmembrane domains, but the mRNA is also expected to be destabilized due to nonsense-mediated decay [34]. Consistent with these expectations, we found that *ok3473* prevents *gem-1(dx66gf)* from suppressing *gon-2(q388)*, and its phenotype closely resembles that of *dx114*. WormBase (WS238) describes the *catp-6(0)* phenotype as embryonic lethal or sterile, based on the deletion allele, *tm3190*. We obtained the *tm3190* mutation from the Mitani laboratory and found that *tm3190* homozygotes closely resemble *dx114* and *ok3473* homozygotes. Therefore, the assignment of *tm3190* as lethal/sterile evidently is due to the poor growth and low brood size of *catp-6(0)* animals.

In addition to blocking suppression of *gon-2(q388)* by *gem-1(gf)*, elimination of *catp-6* activity also causes an obvious Gro (slow growth) phenotype; postembryonic development takes approximately 20% longer than in wild type. Furthermore, *catp-6(0)* animals are almost always Egl (egg-laying defective). We have not characterized either of these phenotypes in detail, although they are exhibited by both *dx114* and *ok3473*.

### Effects of different genotypes on gonadogenesis

We generated a series of single, double and triple mutant strains to investigate the effects of different genetic combinations on gonadogenesis (Tables 1 and 2). Initiation of gonadal cell divisions was not blocked in either the *catp-6(0)* or *gem-1(0)* single mutants, or in the *catp-6(0); gem-1(0)* double mutant. However, the *catp-6(0)* and *gem-1(0)* mutations clearly enhance the *gon-2(q388)* mutant phenotpe when animals are raised at 20°, the upper range of permissive temperature for *gon-2(q388)* (Table 1). *gem-1(0)* enhances *gon-2(ts)* more strongly than does *catp-6(0)*, and the degree of enhancement is not further increased in the *gon-2(ts); catp-6(0); gem-1(0)* mutant (Table 1). Unexpectedly, *catp-6(0)* exhibits slight suppression of *gem-1(0)* at 23.5° (Table 2, lines 5 vs 6, and 7 vs. 8; see Discussion). The gain-of-function mutation, *gem-1(dx66gf),* appears to exhibit weak suppression of *gon-2(q388)*, even in a *catp-6(0)* background (Table 2, line 6 vs. 9); however, the difference observed is not very large, and the opposite trend is observed at 20°. Possibly, *gem-1(dx66gf)* is more active at 23.5°, or it could be that it simply doesn't have much of an effect compared to *gem-1(+)* in a *catp-6(0)* background.

The *gem-1(dx66gf)* mutation alters a residue located within the last of the twelve transmembrane domains of GEM-1, whereas some of the other *gem-1(gf)* mutations that we isolated are situated within the large cytoplasmic loop situated between transmembrane segments 6 and 7 [23]. Since different alleles of *gem-1* might affect different aspects of *gem-1* regulation, we tested whether *catp-6(0)* can block suppression of *gon-2(ts)* by two of the cytoplasmic loop alleles, *dx69gf* and *dx75gf*. We found that *catp-6(0)* also prevents these alleles from efficiently suppressing *gon-2(q388)* (Tables 1 and 2); however, both of these alleles appear to be able to weakly suppress *catp-6(0)* at 20°, and *dx75gf* also appears to weakly suppress *catp-6(0)* at 23.5°.

### Expression pattern of *catp-6::gfp*

In order to investigate the expression pattern of *catp-6*, we used a modified version of fosmid WRM067B_F08 obtained from TransgeneOme project in which the *gfp* coding sequence is fused to the 3′ end of *catp-6*, separated by a short linker region [28].

**Figure 4:**
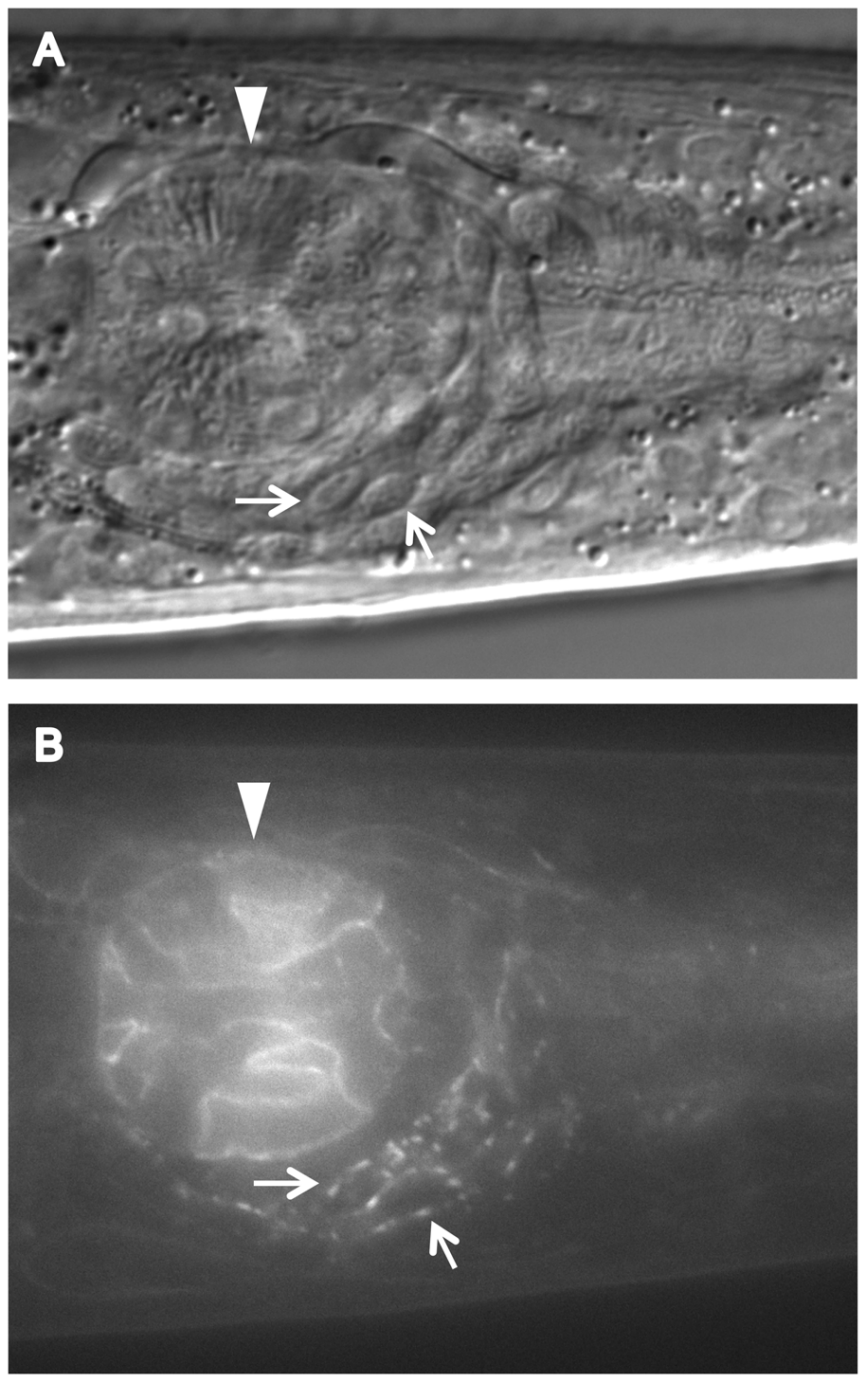
Expression of P*catp-6::catp-6::gfp* in the adult head. A, DIC, B, GFP. Genotype *gon-2(q388); catp-6(ok3473); Ex [Pcatp-6::catp-6::gfp;rol-6(d)]* Arrows indicate two representative neuronal cell bodies. Arrowhead indicates posterior pharyngeal bulb.

**Figure 5:**
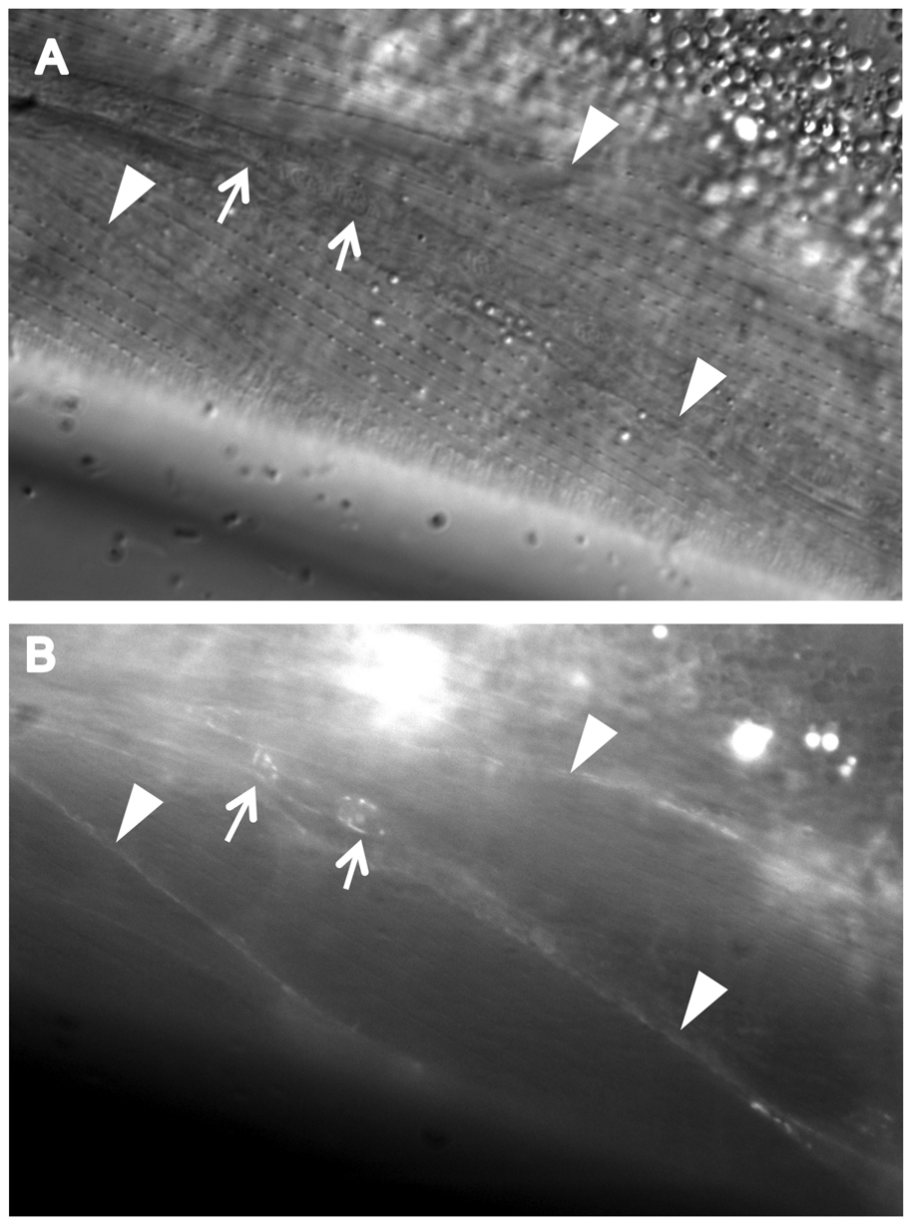
Expression of P*catp-6::catp-6::gfp* in adult body muscle. A, DIC, B, GFP. Genotype *gon-2(q388); catp-6(ok3473); Ex [Pcatp-6::catp-6::gfp;rol-6(d)]*Arrowheads indicate regions where body muscles abut each other. Arrows indicate two neuronal cell bodies. Bright globular patches of fluorescence are autofluorescent gut granules.

**Figure 6:**
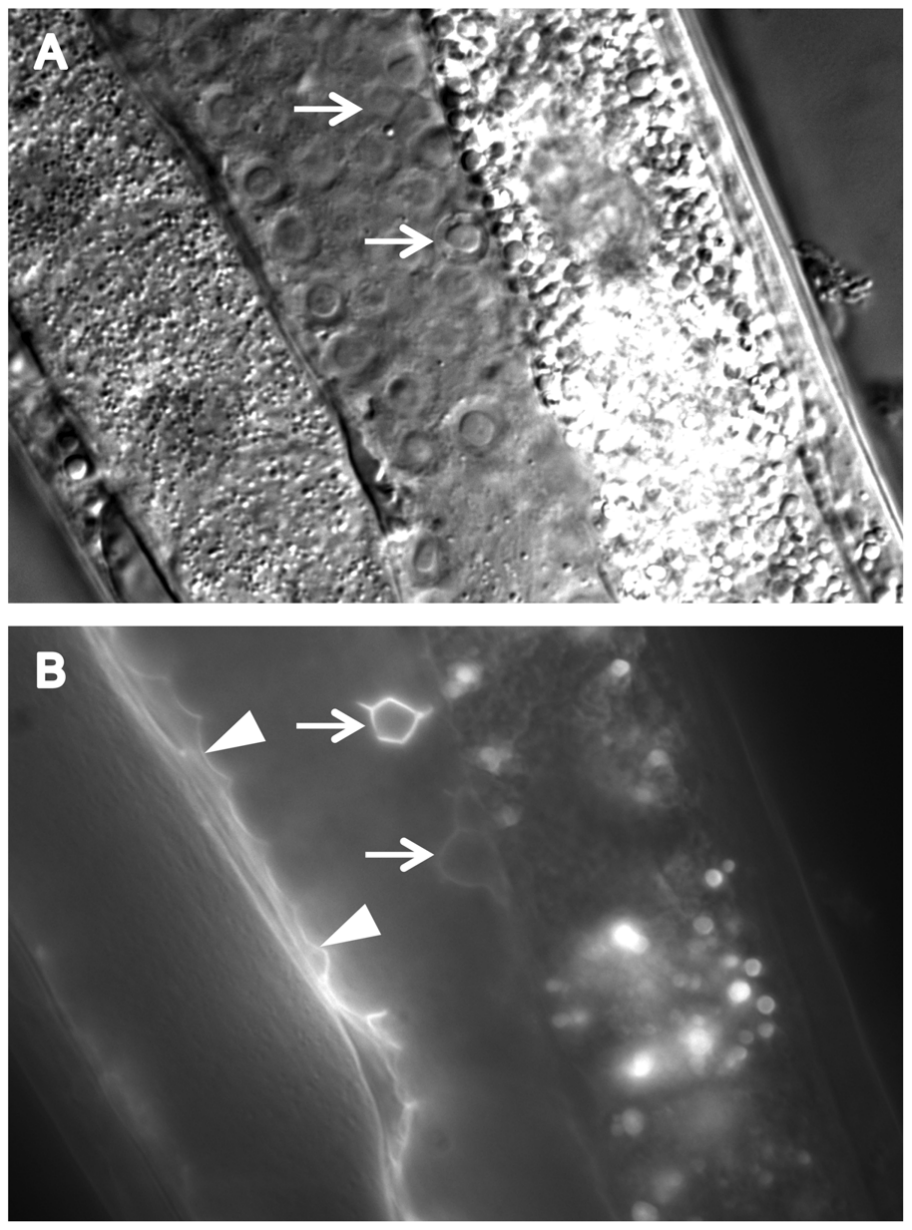
Expression of P*catp-6::catp-6::gfp* in adult gonad. A, DIC, B, GFP. Genotype *gon-2(q388); catp-6(ok3473); Ex [Pcatp-6::catp-6::gfp;rol-6(d)]*. Arrowheads indicate gonadal sheath cell membrane expression. Arrows indicate sheath cell outlining of germ cells undergoing apoptosis. Bright globular patches of fluorescence are autofluorescent gut granules.

**Figure 7:**
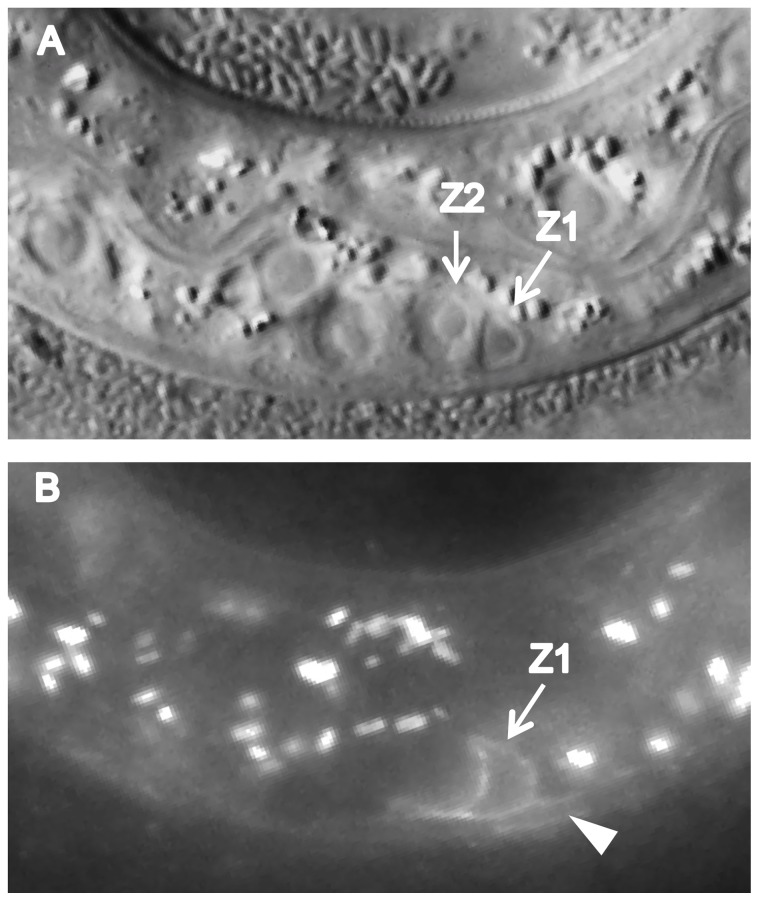
Expression of *catp-6::gfp* in the L1-stage gonad. Genotype *gon-2(q388); catp-6(ok3473); Ex [Pcatp-6::catp-6::gfp;rol-6(d)]*. A, DIC B, GFP. Bright globular patches of fluorescence are autofluorescent gut granules.

We found that CATP-6::GFP is expressed in multiple tissues throughout development. These include a) many neurons in the head and tail (Figure 4), b) all body muscles (Figure 5), c) most pharyngeal cells, particularly in the posterior bulb (Figure 4), d) vulval muscles, e) coelomocytes, f) spermatheca, g) gonadal sheath cells (Figure 6), and h) lateral hypodermis. In many cases, particularly neurons, the fusion protein localizes to cytoplasmic puncta that probably correspond to membranous vesicles (Figures 4 and 5). In other tissues, e.g., pharyngeal cells and gonadal sheath cells, CATP-6::GFP is closely associated with the plasma membrane (Figures 4 and 6). Most significantly with regard to the effect of *catp-6(0)* on gonadogenesis, CATP-6::GFP is associated with the plasma membrane of the somatic gonad precursor cells, Z1 and Z4 (Figure 7).

### CATP-6 expression within Z1 and Z4 rescues gonadogenesis

**Figure 8:**
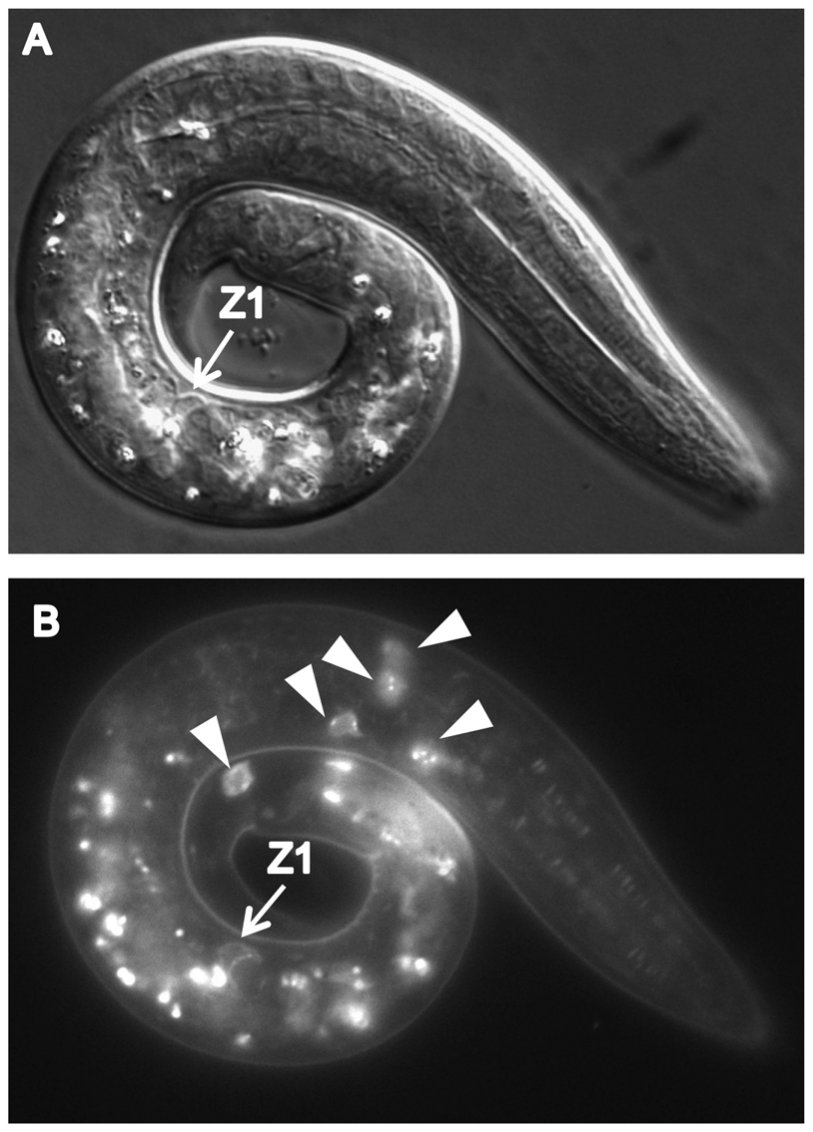
L1-stage expression of P*ehn-3::catp-6::gfp*. A, DIC, B, GFP. Genotype *gon-2(q388); catp-6(0); gem-1(dx66gf)*; *Ex [Pehn-3::catp-6::GFP; rol-6(d)]*. Arroweads indicate neurons in the head and tail. Bright globular patches of fluorescence are autofluorescent gut granules.

**Figure 9:**
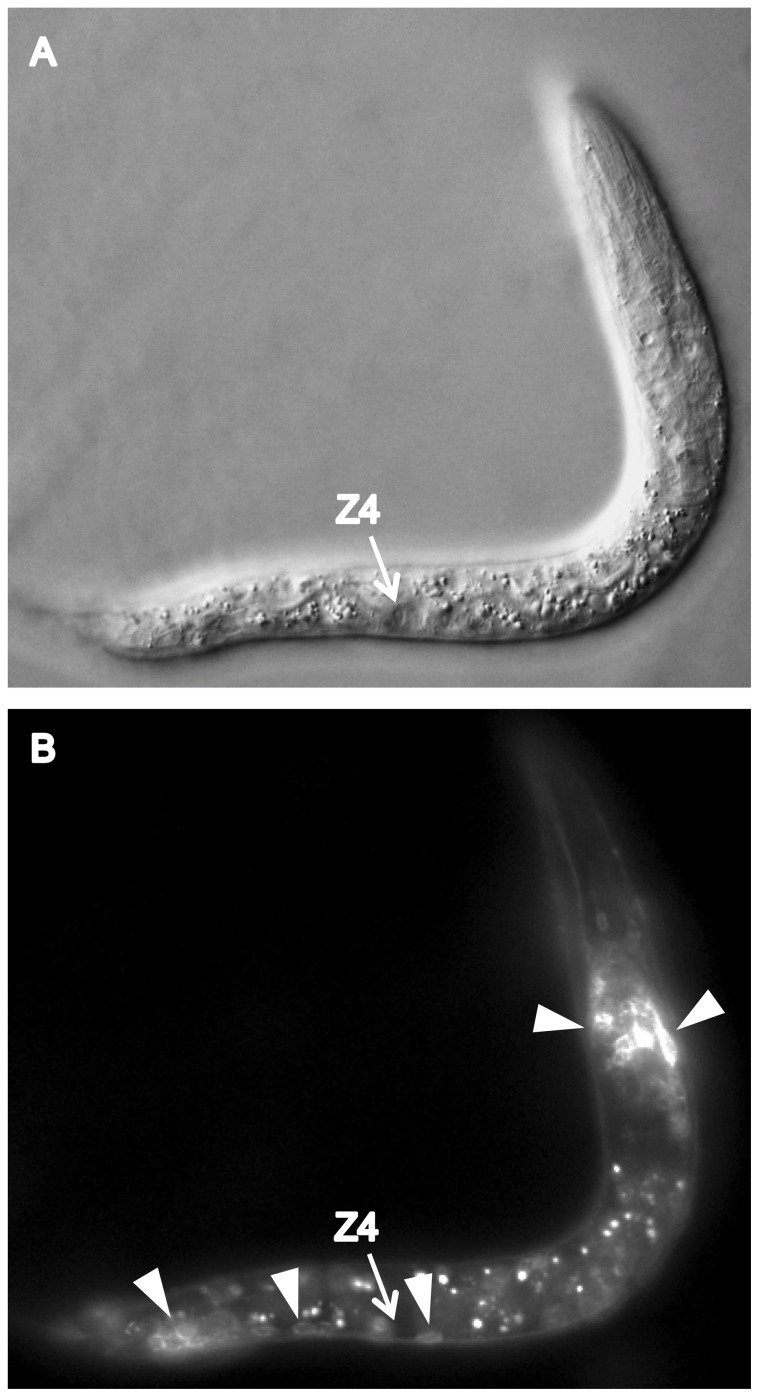
L1-stage expression of P*unc-119::catp-6::gfp*. A, DIC, B, GFP. *gon-2(q388); catp-6(0); gem-1(dx66gf)*; *Ex [Punc-119::catp-6::GFP; rol-6(d)*. Arrowheads indicate representative neurons. Bright globular fluorescence represents autofluorescent gut granules.

**Figure 10:**
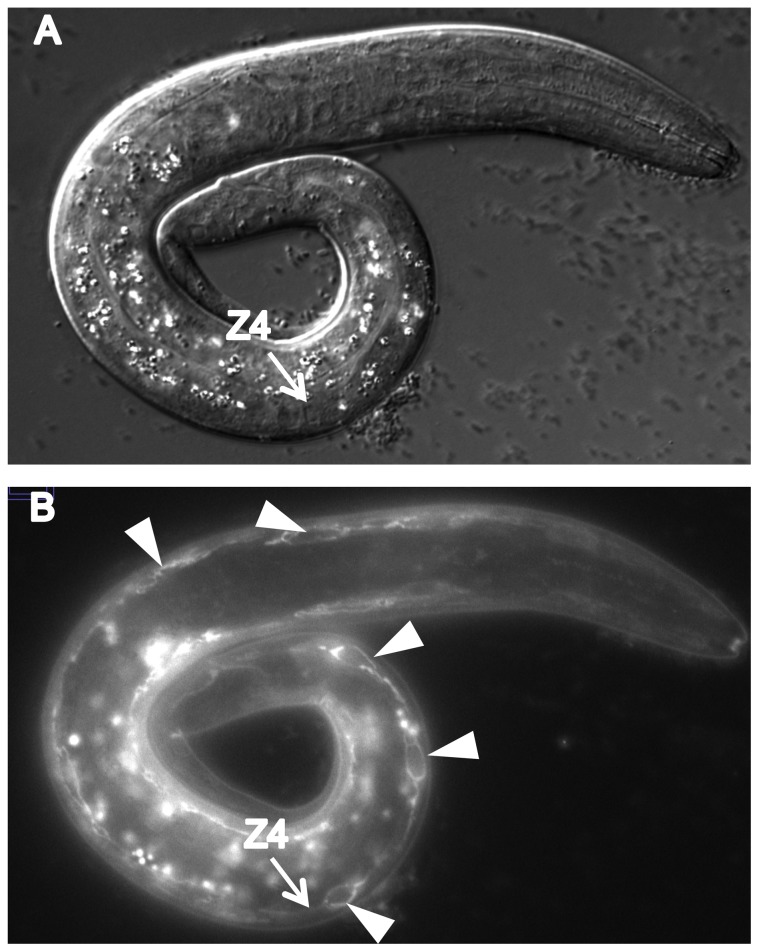
L1-stage expression of P*myo-3::catp-6::gfp*. A, DIC, B, GFP. *gon-2(q388); catp-6(0); gem-1(dx66gf)*; *Ex [Pmyo-3::catp-6::GFP; rol-6(d)*. Arrowheads indicate body muscles. Bright globular fluorescence represents autofluorescent gut granules.

Since *gem-1* and *catp-6* interact genetically, the simplest scenario would be that both genes act within the same cell type, i.e, Z1 and Z4. Indeed, we found that when we used the *ehn-*3 promoter to drive *catp-6::gfp* expression within Z1 and Z4 we were able to rescue the *catp-6(0)* phenotype (Table 3). The *ehn-*3 promoter also drives expression in a small number of neurons in the head and tail region (Figure 8), so it remained formally possible that *catp-6* functions within these cells, rather than the somatic gonad precursors. Therefore, we also tested whether driving *catp-*6 using the pan-neuronal *unc-119* promoter could rescue *catp-6(0)*. Although we did observe widespread expression of *catp-6::gfp* within the nervous system (Figure 9), this did not result in rescue of the *catp-6(0)* phenotype (Table 3). Similarly, when we used the *myo-*3 promoter to drive *catp-6::gfp* in body muscles we did not observe any rescuing activity (Table 3), despite successful expression (Figure 10).

**Table 3:** Effects of transgenes on gonadogenesis at 23.5

	Transgene	Genotype	% Fertile^1^	n
1	*rol-6(d)*	*gon-2(ts); catp-6(0); gem-1(dx66gf)*	4.8	167
2	P*ehn-3*::*catp-6::gfp; rol-6(d)*	*gon-2(ts); catp-6(0); gem-1(dx66gf)*	77.4	106
3	P*catp-6::catp-6::gfp; rol-6(d)*	*gon-2(ts); catp-6(0); gem-1(dx66gf)*	91	100
4	P*myo-3*::*catp-6*::*gfp; rol-6(d)*	*gon-2(ts); catp-6(0); gem-1(dx66gf)*	7.5	226
5	P*gem-1*::*gem-1::gfp; rol-6(d)*	*gon-2(ts); catp-6(0); gem-1(dx66gf)*	55.0	280
6	*rol-6(d)*	*gon-2(ts); catp-6(0); gem-1(dx75gf)*	10.0	68
7	P*ehn-3*::*catp-6::gfp; rol-6(d)*	*gon-2(ts); catp-6(0); gem-1(dx75gf)*	74.2	62
8	P*myo-3*::*catp-6*::*gfp; rol-6(d)*	*gon-2(ts); catp-6(0); gem-1(dx75gf)*	5.6	108
9	P*unc-119::catp-6::gfp*; * rol-6(d)*	*gon-2(ts); catp-6(0); gem-1(dx75gf)*	5.3	131
10	P*gem-1*::*gem-1::gfp; rol-6(d)*	*gon-2(ts); catp-6(0); gem-1(dx75gf)*	47.6	84
11	*Pcatp-6::catp-6::gfp; rol-6(d)*	*gon-2(ts); gem-1(0)*	0.0	111

Genotypes are as in Table 1. Animals were raised and scored as described in Methods. Z test for two population proportions was used to assess signifcance (p<0.05) of differences between different values. Line 1 is significantly different from lines 2, 3 and 5, but not line 4. Line 6 is significantly different from lines 7 and 10, but not lines 8 and 9.

1 Among transgenic (Rol) animals.

### Independent expression and localization of GEM-1 and CATP-6

**Figure 11:**
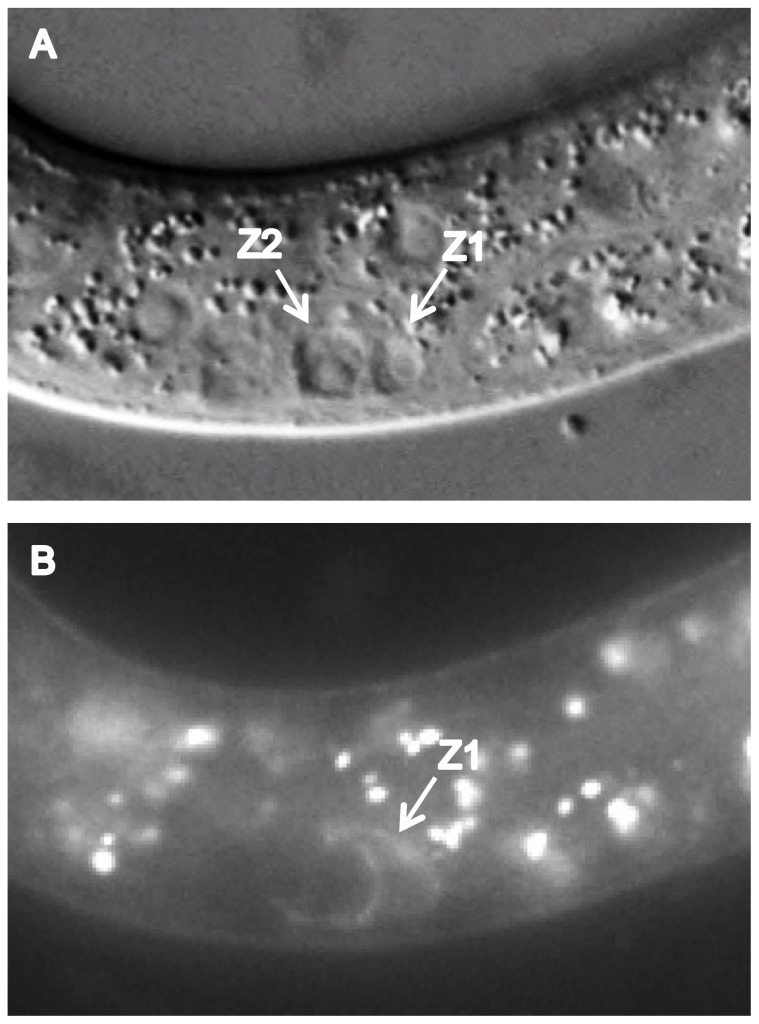
L1 stage expression *of Pcatp-6::catp-6::gfp* in a *gem-1(0)* background. A, DIC, B, GFP. Genotype *gon-2(q388); gem-1(bc364)*; *Ex [Pcatp-6::catp-6::gfp; rol-6(d)]*.

**Figure 12:**
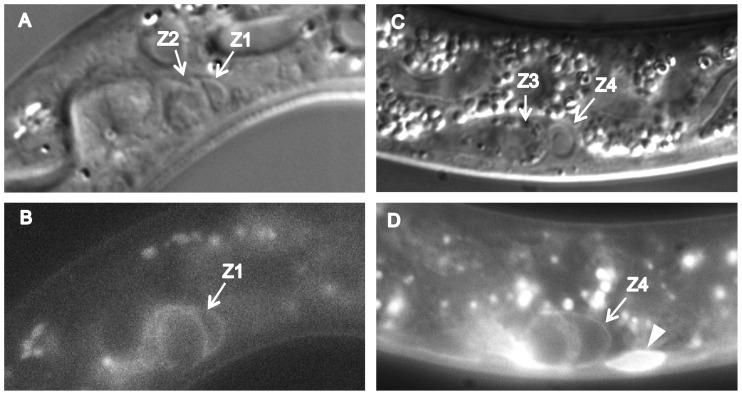
Expression of *gem-1::gfp* in the first larval stage gonad. A and C, DIC, B and D, GFP. A, C, Genotype *gon-2(q388); gem-1(bc364); Ex [gem-1::gfp; rol-6(d)]*. B, D, Genotype *gon-2(q388); catp-6(ok3473); gem-1(dx66gf)*; *Ex [gem-1::gfp; rol-6(d)]*. Arrowead in D indicates overexposed body muscle membrane fluorescence. The animal in panels C and D has more gut granules and a larger nucleolus in Z4, because it is later in the L1 stage than the animal in A and B. Bright globular patches of fluorescence are autofluorescent gut granules.

Since *gem-1(0)* and *catp-6(0)* each enhance *gon-2(ts)* (Tables 1 and 2), their actions could potentially be explained by a simple regulatory relationship in which one gene acts upstream of the other. Given that each gene encodes a membrane protein expressed within Z1 and Z4, one simple possibility would be that one of the proteins acts to recruit the other to the plasma membrane. We tested this possibility by examining the expression/localization of GEM-1::GFP in a *catp-6(0)* background, and CATP-6::GFP in a *gem-1(0)* background. We found that GEM-1::GFP associated normally with the plasma membrane of Z1 and Z4 in a *catp-6(0)* background (Figure 11), as did CATP-6::GFP in a *gem-1(0)* background (Figure 12). Therefore, neither protein is strictly dependent on the activity of the other in terms of expression or subcellular localization. However, since each fusion construct is present on an extrachromosomal array, we cannot fully exclude the possibility that normal regulatory constraints might be overwhelmed by overexpression from the transgene. Furthermore, higher resolution imaging would be necessary to detect subtle changes in subcellular protein localization.

### Effects of overexpression of CATP-6 and GEM-1

Using the transgenic strains described above, we tested whether expression of CATP-6::GFP could bypass the requirement for *gem-1(+)*. As discussed above, since the fusion protein is encoded on a multicopy extrachromosomal array, it is likely that its expression level exceeds that of the wild type CATP-6 protein. However, we observed no rescue of the *gem-1(0)* phenotype in the transgenic animals (Table 3). This is in contrast to our observations regarding GEM-1::GFP. In this case, we did observe rescue of the *catp-6(0)* phenotype, suggesting that high level expression of GEM-1 can bypass the requirement for CATP-6 (Table 3).

## Discussion

Our observation that *catp-6* activity is required in order for *gem-1(gf)* to suppress *gon-2(lf)* could potentially be explained by various different scenarios. Based on the known ability of the prokaryotic P-type ATPases, MgtA and MgtB to mediate Mg^2+^ uptake, a simple model for the relationship between GEM-1 and CATP-6 would be that GEM-1 exerts some form of positive regulation on CATP-6, which then pumps Mg^2+^ into the cytoplasm of Z1 and Z4. Although this model is attractive because of its simplicity, several lines of evidence suggest that it is probably not correct. First, the most similar eukaryotic P-type ATPases, ypk9 and ATP13A2, are thought to pump divalent cations- into intracellular vesicular compartments (i.e., out of the cytoplasm). Second, with regard to gonadogenesis, the phenotype of *gem-1(0)* is more severe than that of *catp-6(0)*. Third, neither expression nor subcellular localization of CATP-6::GFP is strictly dependent on GEM-1 activity. Fourth, overexpression of GEM-1::GFP is able to bypass *catp-6(0)*. However, none of these individual arguments is completely definitive: In the first case, neither the ion specificity nor the directionality of transport of P5B ATPases has been directly measured. In the second, third and fourth cases, it is possible that one of the paralogous genes, *catp-5* or *catp-7*, is able to provide compensatory activity in the absence of *catp-6* function.

**Figure 13:**
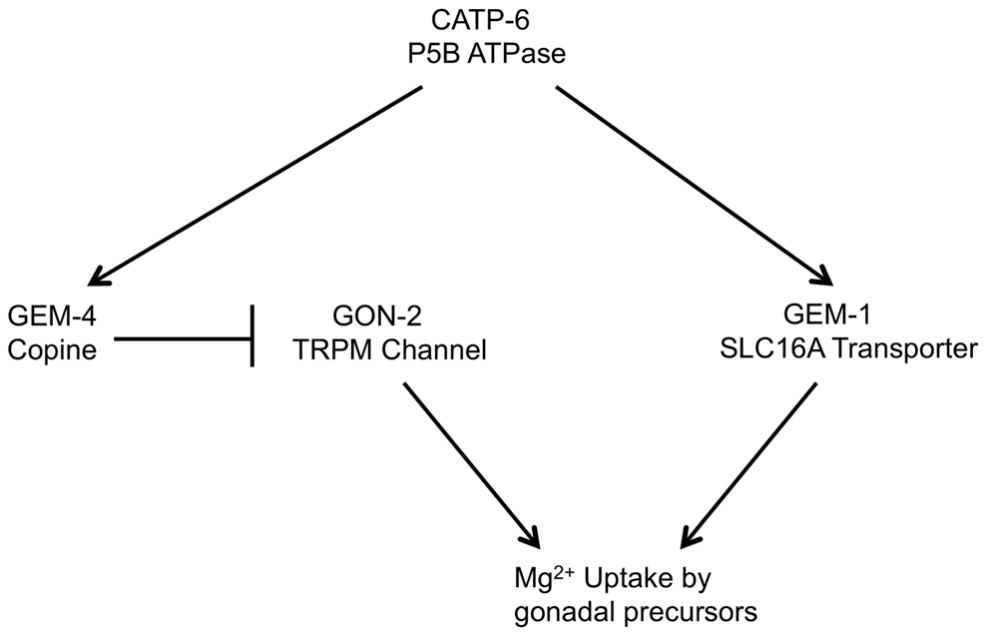
Model of possible regulatory relationships between CATP-6, GEM-1, GEM-4 and GON-2. Arrows indicate positive regulation and “roadblocks” indicate negative regulation.

Since GEM-1 appears to be capable of functioning even in the absence of CATP-6 activity, we consider it most likely that CATP-6 acts as a positive regulator of GEM-1 that is dispensable under conditions of excess GEM-1 protein (Figure 13). Possible explanations for such a positive regulatory interaction include, but are not limited to, the following: a) CATP-6 could promote the proper targeting of GEM-1 to the plasma membrane, either via a direct protein-protein interaction, or by regulating vesicular trafficking, b) CATP-6 activity could be required to maintain normal lysosome function; dysfunction of lysosomes could lead to inappropriate sequestration and/or degradation of GEM-1, c) CATP-6 could pump Mg^2+^ into an intracellular compartment from which it is later released by GEM-1. In this case, overexpression of GEM-1 on the plasma membrane might permit sufficient Mg^2+^ import to render access from intracellular compartments unnecessary, d) CATP-6 could act as a polyamine importer, or positively regulate a polyamine transporter, (as proposed for CATP-5), and polyamines could promote GEM-1 activity, e) CATP-6 and GEM-1 could directly interact to form a Mg^2+^ importer, with CATP-6 acting as a non-essential, regulatory subunit.

*gon-2(lf); gem-1(0)* hermaphrodites exhibit a highly penetrant gonadogenesis defect that is weakly suppressed by inactivation of *catp-6*. One possible explanation for this suppression is that CATP-6 might also be a positive regulator of GEM-4 (Figure 13). We previously found that inactivation of GEM-4 partially suppresses the gonadogenesis defect of *gon-2(lf); gem-1(0)* animals, possibly by relief of negative regulation of GON-2. Since GEM-4 also associates with the plasma membrane of Z1 and Z4, CATP-6 could potentially affect GEM-4 function either by a direct interaction, or indirectly via alteration of vesicular trafficking. A comparable situation might also exist in the case of CATP-5, where it could be that this protein exerts its effects on polyamine uptake by regulating the association of a separate tansporter protein with the plasma membrane.

Although we detect CATP-6::GFP in close association with the plasma membrane in some tissues, we cannot be certain that the protein is actually located within the plasma membrane. For example, in the case of Z1 and Z4 the fluorescence pattern of CATP-6::GFP (unlike that of GEM-1::GFP) is often discontinuous along the periphery of the cell. Thus, it remains possible that the protein localizes to vesicles that are just beneath the plasma membrane. Furthermore, it is possible that CATP-6 localizes to both plasma membrane and vesicular compartments and that the relative distribution differs between cell types. Higher resolution microscopy will be necessary to resolve this issue.

Clearly, further work must be done in order to distinguish between the many possible models for the interactions between CATP-6 and GEM-1, as well as to resolve the issues of transport specificity of each of these proteins. In the case of GEM-1, we have no direct evidence that the protein functions as a transporter; however, its similarity to known transporters makes it highly likely that this is the case. For CATP-6, our findings that one of the alleles that we isolated (*dx112*) alters a residue that is predicted to directly interact with substrate and another (*dx114*) affects a residue expected to be critical for enzymatic activity suggest that this protein is very likely to function as a typical P-type ATPase transporter protein. Although not unexpected, this was not a foregone conclusion, e.g., Ruaud and Bessereau (2007) have shown that *C. elegans* CATP-1, a type IIC P-type ATPase, has important functions that are independent of its catalytic/transport activity.
